# Balancing Trade‐Offs in Patient and Public Involvement and Engagement in Rapid Evaluation: Reflections and Lessons

**DOI:** 10.1111/hex.70749

**Published:** 2026-07-01

**Authors:** Pei Li Ng, Raj Mehta, Angus I. G. Ramsay, Naomi J. Fulop, Jenny Shand

**Affiliations:** ^1^ Research Department of Behavioural Science and Health, Institute of Epidemiology and Healthcare University College London London UK; ^2^ Public Contributor London UK; ^3^ Department of Clinical Educational and Health Psychology University College London London UK

**Keywords:** health and social care research, impact on research, patient and public involvement, user involvement

## Abstract

**Background:**

Patient and public involvement and engagement (PPIE) is recognised as essential to high‐quality health and care research. However, there is limited guidance on how to sustain meaningful involvement in rapid evaluations characterised by compressed timelines and shifting priorities.

**Objective:**

To reflect on how PPIE developed within the NIHR Rapid Service Evaluation Team (RSET) and to identify practical lessons for navigating tensions between influence, feasibility, and equity in time‐pressured contexts.

**Methods:**

This practice‐based critical reflection draws on internal documentation, shared reflections, and evaluation activities from 13 rapid evaluations (2018‐2026). Using the International Association for Public Participation (IAP2) Spectrum as a guiding tool, we mapped RSET's PPIE approach across three phases: Early Phase, Developing Maturity, and Current Position, highlighting trade‐offs in practice.

**Results:**

Early involvement was meaningful but ad hoc, constrained by limited infrastructure and resources. As the programme matured, more structured yet flexible approaches were introduced, including a standing PPIE panel, tailored involvement aligned to lived experience, and earlier engagement at key decision points. Involvement practice required ongoing negotiation of trade‐offs, including Depth vs Speed, Inclusivity vs Practicality, Consistency vs Flexibility, Relationship‐building vs Time, and Value vs Feasibility.

**Conclusions:**

Meaningful PPIE in rapid evaluations is achievable when involvement is planned intentionally, responsive to context, and supported by strong collaboration and communication. Rather than pursuing full co‐production, effective involvement focuses on relationships and influence where it adds most value, offering transferable lessons for involvement practice in fast‐paced research settings.

**Patient or Public Contribution:**

This paper examines PPIE practice. Public contributors were involved in the programme, contributed to the development of PPIE approaches and practices, and reflected on their experiences. One co‐author was a public contributor who helped shaped the interpretation and lessons presented in this paper.

## 1 Introduction

Patient and public involvement and engagement (PPIE) is increasingly recognised in UK health and social care research as both an ethical expectation and a driver of improving research quality. It ensures studies address the concerns and priorities of patients and the public while promoting relevance, accountability, and equity [1, 2, 3, 4, 5]. Frameworks such as the UK Standards for Public Involvement [6] and NIHR INVOLVE [7] promote inclusive, respectful, and purposeful public collaboration. Internationally, several countries have developed similar frameworks emphasising partnerships with patients and communities to shape research and care delivery [8, 9, 10, 11, 12].

Despite this maturation of guidance, embedding meaningful PPIE remains challenging. Persistent issues of representation [13], power dynamics [5, 14, 15, 16], time and resources for relationship‐building [15, 16, 17, 18, 19], and unclear expectations [16, 17, 18] are well documented. These challenges may be amplified in rapid evaluations, where studies are delivered within compressed timelines, accelerated set‐up processes, and rapidly changing policy or service priorities. Early involvement is therefore often particularly challenging in this context, as contributors are asked to shape research on innovations or services that are still evolving [20, 21]. These well‐documented features may limit opportunities to build trust or involve diverse voices early in shaping research design. Rapid turnaround expectations may also create practical burdens for public contributors and unintentionally exclude those unable to engage within short timeframes.

Much existing PPIE guidance is developed within assumptions of traditional research conditions, including stable timelines, extended planning, and iterative co‐design cycles. These conditions allow time and space for relationship building and progressive refinement of involvement activities. However, they may not align with the realities of rapid evaluation, which is designed to generate timely evidence in fast‐moving health and care settings [22, 23]. As a result, established PPIE models may require adaptation to remain feasible while still enabling meaningful influence. Without careful adaptation, PPIE risks becoming aspirational rather than practical: introduced too late, done superficially, or lacking genuine influence, which can lead to tokenism [24] and undermine research relevance and quality [19].

While methodological considerations for rapid evaluation are increasingly discussed [20, 25, 26, 27, 28], guidance on how PPIE is operationalised in rapid research programmes remains limited. In particular, little is known about how teams determine where involvement has greatest influence, how trade‐offs are negotiated, or how PPIE evolve across sustained programmes. Building on Smith et al. [20], who highlighted trade‐offs such as rigour versus rapidity, this paper examines how such trade‐offs shape PPIE practices as the programme matures.

This paper draws on the experience of the National Institute for Health and Care Research (NIHR) Rapid Service Evaluation Team (RSET) [29], which has delivered over 13 rapid evaluations since 2018. Using the International Association for Public Participation (IAP2) Spectrum [30] to map levels of public involvement, we examine the programme's progression and provide practical lessons for negotiating what is possible, fair, and influential in rapid evaluation contexts.

We propose that meaningful PPIE in rapid evaluation does not necessarily require full co‐production, which involves contributors sharing power as equal partners at all stages [31]. Co‐production broadly refers to researchers and the public working together to achieve a shared outcome, though its meaning varies across contexts [32, 33]. While full co‐production is an important ideal, in practice it can be challenging to achieve in both rapid and non‐rapid research, although the constraints are often more pronounced in rapid contexts, due to time, resources, and study design. In rapid evaluation settings, we instead adopt a fit‐for‐context approach that focuses on strategically integrating public perspectives where they can have the greatest impact, within evolving priorities and limited timelines.

## 2 Context

RSET is a multidisciplinary and generalist team delivering rapid evaluations of health and care services to support timely evidence‐based decision‐making nationally [20]. Its remit spans diverse settings, including primary and secondary care, social care, and prisons. Operating under the ‘4 Rs’ concept ‐ *Rapidity, Responsiveness, Relevance, and Rigour*, rapid evaluations aim to deliver credible findings within shorter timeframes than traditional research [20]. Key characteristics include accelerated planning, adaptive study design, and real‐time formative feedback [20, 26, 34], which can create structural tensions for PPIE [20].

Emerging literature highlights common challenges of PPIE in rapid settings: short timelines restricting trust‐building [20, 35, 36], risks of tokenism [35], early clarity on roles and expectations [20, 37], and difficulties recruiting relevant lived experience especially in novel or highly specialised contexts [21, 34, 35, 38]. These findings emphasise that, without adequate resources and adaptive strategies, PPIE risks being superficial, limiting contributions to study design, equity, and relevance.

Despite these insights, evidence on how PPIE evolves over sustained rapid research programmes remains limited. Key gaps include: (1) How involvement evolves beyond single projects; (2) How organisational capability and infrastructure for PPIE mature; and (3) How teams navigate practical constraints and make transparent trade‐offs. To address these gaps, we examine how PPIE can be embedded in rapid evaluation, what is feasible, and how trade‐offs can be managed to maintain relevance, timeliness, and responsiveness [34].

## 3 Our PPIE Journey Through a Maturity Lens

This paper draws on 7 years of practice‐based experience (2018‐2026) within the RSET programme and is informed by multiple sources of organisational learning. These include internal documentation (e.g. meeting notes, PPIE strategy, progress reports, and evaluation records), contributors’ feedback, and ongoing reflective discussions among researchers and public contributors involved in the programme. Reflections occurred both informally through routine collaborative working across individual evaluations and more formally through programme‐level discussions within PPIE governance structures.

As authors embedded within the programme, we adopt a critical reflective stance in interpreting these experiences, recognising both the strengths and limitations analysing our own organisational practices over time. One public contributor co‐author was involved throughout the development of this manuscript, including conceptualisation, interpretation, drafting, and revision. In addition, perspectives from the PPIE Panel (8 members), along with wider PPIE activities (approximately 25 public contributors across workshops and engagements), informed the analysis through feedback, discussions and reflective sessions. However, not all contributors were directly involved in reviewing the manuscript.

**Figure 1:**
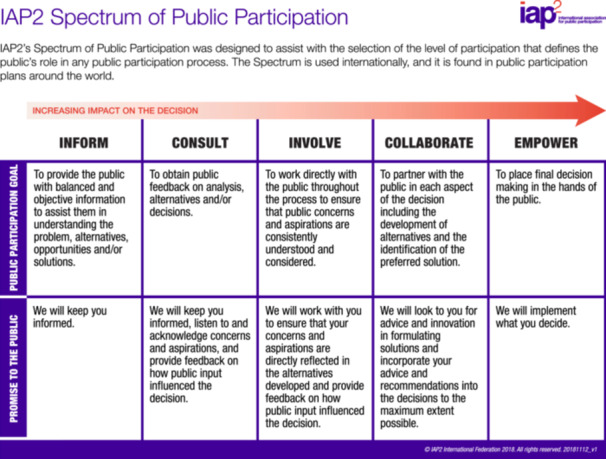
IAP2 spectrum of public participation. ©International Association for Public Participation www.iap2.org.

**Figure 2:**
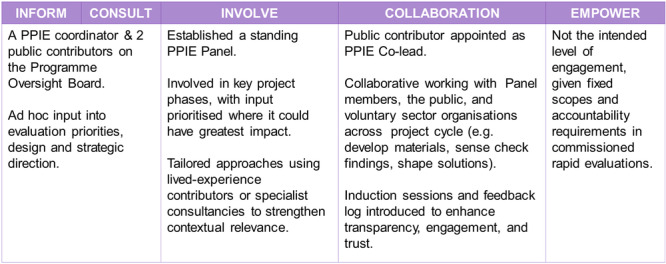
RSET's adaptive PPIE journey across the IAP2 spectrum.

We use the IAP2 Spectrum [30] (Inform, Consult, Involve, Collaborate, Empower; see Figure 1) as a guiding tool to map the evolution of our PPIE approach over time (Figure 2), rather than implying progression toward an ideal end point.

### 3.1 Early Phase (2018–2020): Foundation Involvement (Inform–Consult)

From inception, a PPIE specialist and two contributors were appointed to the Programme Oversight Board to advise on evaluation priorities and strategic direction. We also engaged with a PPIE group from another research programme, and individual evaluations involved contributors with relevant lived experience through ad hoc advisory groups, for example in an outpatient service innovation study [39].

During this phase, involvement mainly fell at the Inform and Consult levels, it was ad‐hoc and varied across projects. Despite limited structures, relationships formed with contributors, who valued being listened to and provided insights that directly shaped evaluation priorities and outputs.

For example, in the early stages of RSET, contributors highlighted the importance of exploring barriers to accessing services across patient pathways and experiences. This input led to a stronger emphasis on access‐related issues within subsequent evaluations, including a greater focus on inequalities, referral pathways, service navigation, and unintended consequences in evaluation questions and topic guides. This was reflected in the evaluation of COVID‐19 remote home monitoring services, where research questions and analyses explicitly examined digital exclusion and associated health inequalities [40]. In another discussion, contributors encouraged the team to look beyond health sector and consider how other industries organise and deliver services, for example comparing centralised and outsourced models. This prompted the study team to explore whether organisational models could inform the development of attributes to be used in a discrete choice experiment (DCE), a survey‐based method used to elicit preferences. Contributors also influenced recruitment approaches by recommending clearer wording and more appropriate outreach channels for engaging underserved groups, including engagement through relevant community organisations and charities. These suggestions shaped the design of study advertisements and recruitment strategies across several evaluations.

The departure of the PPIE coordinator in 2019 highlighted vulnerabilities in capacity and continuity. Limited staffing and budgets shaped how involvement was prioritised, resulting in more selective integration of PPIE across the programme and projects.

This ‘initial involvement’ was authentic and meaningful, enabled through relatively light supporting infrastructure, illustrating how rapid contexts shape the distribution and depth of influence depending on organisational readiness and structural support. This reflects a clear trade‐off between depth of involvement and compressed timelines.

### 3.2 Developing Maturity Through Structures and Readiness (2020–2023: Inform–Consult–Involve)

As evaluation volume increased, a structured but flexible PPIE approach became necessary. A PPIE strategy was co‐developed with contributors in 2020 to guide the approach for the remainder of the programme. A PPIE Panel was established in 2021, expanding contributors from two to four, marking a transition to the Involve level. The expanded panel brought new perspectives, laid the foundation for long‐term relationship building, and supported contributors to build familiarity with rapid evaluations processes.

Contributors began playing more active roles in shaping study design, though fully embedding PPIE across all stages remained constrained by time and competing priorities. To maximise impact, the team prioritised early‐stages input, such as reviewing patient‐facing materials, piloting questionnaires, and refining interview topic guides. Later‐stage involvement was sometimes scaled back when data collection delays compressed analysis and write‐up, with decisions discussed and agreed collaboratively. This pragmatic approach balanced meaningful input with responsiveness under time and resource constraints.

Given the diversity of evaluation topics, the PPIE approach became increasingly tailored. In some evaluations, the established Panel provided relevant perspectives: in the prehospital triage pilot study [41], panel members were involved early in shaping study design and materials. Engagement was further supplemented by a stroke survivor at key phases. All contributors co‐authored the final report. This model balanced panel continuity with targeted lived experience to strengthen research relevance.

In other contexts, such as the prison peer support evaluation [42], partnering with a specialist consultancy enabled rapid engagement with individuals with firsthand experience of imprisonment and social care. This targeted approach ensured relevance and sensitive involvement, helped refine participant‐facing materials and interpreting findings appropriately, despite tight timelines.

These experiences illustrate adaptive strategies balancing continuity, inclusivity, and context‐specific relevance.

### 3.3 Our Current Position: Collaborate (2023–to Date)

**Figure 3:**
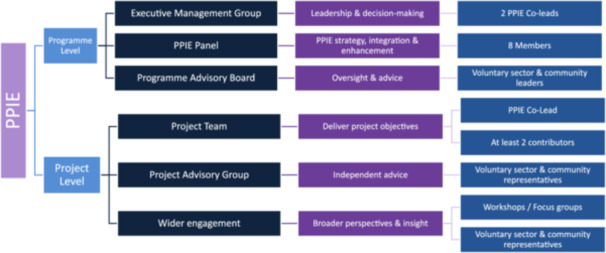
RSET (2023–2028) multi‐tiered PPIE structure.

Building on lessons from the previous funding cycle, the RSET 2 funding bid offered the opportunity to redesign our PPIE approach. In the current cycle (2023‐2028), our approach has matured to the Collaborate level. Together with PPIE Panel, we co‐designed a strengthened PPIE strategy and a multi‐tiered structure (Figure 3), guided by the UK Standards [6], which provided a robust foundation for principles. However, applying these standards in rapid evaluations required careful adaptation to account for constraints. Day‐to‐day operational and methodological decisions remain with the research team, while contributors are now engaged earlier, with clearer roles, and consistent opportunities for input. The PPIE budget was increased substantially compared to the previous cycle, with a standard benchmark introduced for each project to flexibly scale resources according to needs.

Positioning ourselves at the Collaborate stage reflects a commitment to meaningful collaboration, balanced with practical constraints that require researchers to retain final decision‐making authority. Compressed timelines and fixed scopes mean contributors can shape *how* research is done but cannot redefine *what* must be delivered. This limits the feasibility of full co‐production. However, the IAP2 Spectrum should not be interpreted as implying that higher levels of participation (e.g. Empower) are always preferable. In rapid evaluation contexts, the appropriateness of involvement is better judged by its relevance, timeliness, and influence rather than progression along the spectrum. Even with these constraints, contributors can still bring significant influence by strengthening contextual sensitivity and integrating public perspectives. By focusing involvement where it can have meaningful impact, this approach balances influence, feasibility, and equity. Clear boundaries help manage expectations and support honest conversation about roles and power.

#### 3.3.1 Programme‐Level Structures

PPIE leadership is jointly held by a contributor and the programme manager (also co‐investigators), both sitting on the Executive Management Group. This embeds public voice within governance and signals a visible organisational commitment to PPIE. Importantly, responsibility for involvement does not sit solely on PPIE leads, senior researchers also act as internal advocates, reinforcing PPIE as a collective ethos. Dedicated staffing, ring‐fenced budgets, and leadership advocacy provide the infrastructure needed to support PPIE at pace.

Led by the PPIE co‐leads, the PPIE Panel expanded its membership through targeted recruitment of groups that had previously been under‐represented in the programme involvement activities, including younger adults, minority ethnic communities, and individuals with caring responsibilities. The aim of this recruitment was to broaden the range of perspectives and lived experience, rather than to achieve statistical representation. The recruitment process involved two panel members shortlisting applicants and conducting interviews, as well as making selection decisions. This broadened the diversity of lived experience within the panel and strengthened the team's ability to mobilise contributors early into new evaluations, helping to address the challenge of involvement readiness. The panel was designed as a permanent programme‐level structure, complemented by flexible project‐level involvement to provide additional perspectives where required. This dual structure supports early involvement while allowing flexibility to respond to different topic areas and populations. Contributors described a growing sense of belonging and continuity, which is an important enabler in contexts where trust‐building time is limited [15]. The Programme Advisory Board now also comprises leaders from health care delivery and voluntary organisations with PPIE expertise, acting as ‘critical friends’ to provide external scrutiny and strengthen practice.

#### 3.3.2 Project‐Level Collaboration

Regular engagement is vital for expediting relationship‐building and integrating actionable insights in real time. Induction sessions with clearly defined roles help clarify expectations. Contributors are now engaged more intensively at early stages, including attendance at project meetings during discovery and scoping phase, where their input help shape research questions, study design, and project materials (e.g. recruitment advert). This early involvement is particularly important because it helps ensure that the research focuses on questions and outcomes that matter to people with lived experience. As projects move into data collection phase, project meetings tend to focus on operational progress (e.g. recruitment status), with limited opportunity for interpretive discussion. In these phases, involvement becomes more targeted through dedicated meetings, avoiding unnecessary burden while providing a safe space to share reflections that may not feel appropriate in formal project meetings. This reflects an intentional prioritisation of involvement at stages of greatest influence, balancing value and feasibility.

Topic‐specific workshops and collaboration with voluntary sector organisations provide wider perspectives and flexible mechanisms for those unable to commit long‐term or to fill specific perspective gaps. For example, in a maternity service evaluation [43], we applied lessons from the prison evaluation [42] by partnering with a national charity to co‐design key elements of the study. We also held workshops with families from underserved groups to sense‐check study materials and identify blind spots. This combined approach, drawing on voluntary sector input and targeted workshops, was chosen based on project needs, timelines, and the importance of capturing diverse perspectives to ensure research outputs reflected those most affected.

We also introduced a simple but effective “You said, we did” feedback log, suggested by a Panel member, to improve transparency and close feedback loops. This mechanism is integrated across all programme and project meetings to document how contributors’ views influence decisions and explains when suggestions cannot be incorporated. For example, feedback led to simplifying wordings in patient‐facing materials and recruitment strategies. Contributors reported this promotes openness, fosters trust, and makes collaboration feel authentic despite the fast‐paced context, which serves as a clear indicator of meaningful PPIE [44].

### 3.4 Maturity in a Rapid Research Setting: A Person‐Centred Perspective

Our maturity journey has been iterative, shaped by shifting priorities and continual experiential learning. In rapid contexts, sustaining meaningful involvement depends as much on relational continuity and readiness as on structures and processes. Contributors familiar with the team's ways of working can offer timely and honest insights, supporting effective collaboration at pace.

A key learning was the importance of a person‐centred approach to involvement. Decisions about when and how to engage contributors must balance project needs with contributors’ interests, capacities, and availability. Contributors described valuing flexibility and choice in how they were involved. This allowed them to engage more deeply in areas aligned with lived experiences, or step back temporarily when personal circumstances required. These fluctuations create ongoing trade‐offs between consistency and flexibility, rigid approaches would risk undermining inclusivity and feasibility. To build resilience, we ensure at least two contributors are involved in each evaluation to maintain continuity if one is unavailable.

Accessibility is prioritised through alternative formats and adapted meeting times to reduce participation barriers without slowing project timelines. Contributors emphasised that being listened to and having their needs responded to helped foster trust and made involvement feel worthwhile.

Based on reflection, PPIE maturity in rapid evaluation is about appreciating the value of PPIE, planning it intentionally, and developing a shared understanding of what is feasible and meaningful. It emphasises person‐centred collaboration under time pressure, prioritising strong relationships over task completion, rather than aiming for full co‐production or the Empower level on the Spectrum.

### 3.5 Discussion: What This Means for PPIE in Rapid Evaluation

Rapid evaluations’ tight timelines, shifting scopes, and limited resources require adaptive approaches to keep involvement meaningful. Central to this is a shared purpose to improvement between contributors and researchers, with flexible approaches ensuring involvement remains impactful.

Our reflection highlights challenges and tensions rarely made explicit in the PPIE literature. Frameworks such as the UK Standards [6] and Agile Co‐production and Evaluation (ACE) [45] provide useful principles, however, applying them in rapid contexts requires careful adaptation. For instance, influence is not evenly distributed across the research lifecycle. Early‐stage involvement in shaping evaluation questions, recruitment strategies, and materials consistently had the greatest impact, whereas later‐stage engagement (e.g. writing and dissemination) was more vulnerable to compressed timelines and limited resources.

Rather than treating these patterns as shortcomings, RSET approached them as design challenges requiring explicit negotiation. Decisions about where to concentrate involvement, and where to scale it back, were made transparently through open discussion with contributors, PPIE co‐leads (including a public contributor co‐lead), and project leads. There was usually agreement on where involvement would have most impact, but this was not always unanimous, particularly around the timing and depth of involvement under rapid evaluation conditions. In these cases, constraints and implications were discussed openly and decisions were reached by project leads in consultation with PPIE co‐leads, drawing on their shared assessment of what was feasible within available timescales and resources. Project leads retained final responsibility for methodological and delivery requirements, but decisions were informed by these discussions. Contributors valued the transparency of this process, even where not all preferences could be accommodated. This approach was critical to trust‐building and helped avoid erosion of relationships [19] that can occur when involvement is reduced abruptly or without explanation.

#### 3.5.1 Negotiating Trade‐Offs in Practice

Trade‐offs between what is desirable and what is feasible were a central feature of PPIE in rapid evaluation. Key tensions include Depth vs Speed, Inclusivity vs Practicality, Consistency vs Flexibility, Relationship‐building vs Time, and Value vs Feasibility. Table 1 summarises these tensions and the strategies RSET adopted in response.

**Table 1:** Key tensions and strategic trade‐offs in embedding PPIE within rapid evaluations.

Tensions in rapid context	Reflective question	Strategy (with examples/impact)
Depth vs speed	*How much involvement is possible without delaying delivery?*	Prioritised early and high‐impact stages, using focused methods (e.g. targeted consultation or workshops to shape recruitment strategies and interview guides), supporting timely delivery and relevant input.
Inclusivity vs practicality	*How can diverse perspectives be included meaningfully within time and resource constraints?*	Combined the standing PPIE Panel with supplementary engagement through trusted partners and community organisations (e.g. maternity charity, prison research consultancy) to improve equity and contextual sensitivity.
Consistency vs flexibility	*What should be standardised across projects, and where is flexibility needed?*	Introduced a standard PPIE budget benchmark with flexibility to scale involvement where experiential insight is especially critical, promoting fairness while remaining responsive.
Relationship‐building vs time	*How to build trust efficiently under time constraints?*	Invested early in relationship‐building through clear expectations, regular communication, feedback loops, and a ‘no surprises’ approach (e.g. ‘You said, we did’), strengthening trust and contextual understanding.
Value vs feasibility	*Where is involvement likely to add most value, and where is scaling back justified?*	Made negotiated and transparent decisions about prioritising PPIE in phases with greatest potential impact (e.g. early‐stage input), focusing involvement where it adds most value.

Several cross‐cutting insights emerged. First, valuable PPIE in rapid contexts often prioritised influence over intensity. Focused, time‐limited engagement at key decision points offered meaningful contribution without jeopardising project delivery. Second, fairness and equity were supported through programme‐level consistency and project‐level flexibility, aligning resources where lived experience was most critical. Third, relational work such as setting clear expectations, maintaining regular communication, and providing feedback, were essential for sustaining trust.

Making these trade‐offs transparently reframes how the value of PPIE in rapid evaluations is understood. Rather than assuming that more involvement is always better, or that deviations from idealised models represent failure, our experience suggests that value lies in how thoughtfully trade‐offs are identified, justified, and managed to maximise meaningful impact. Contributors reflected that these trade‐offs could be challenging but appreciated transparent discussion about what was feasible and why.

### 3.6 Strengths and Limitations

This paper provides a longitudinal reflection on PPIE evolution in a sustained rapid evaluation programme, focusing on how trade‐offs are negotiated over time. A key strength is the explicit examination of balancing influence, feasibility, and equity using concrete examples. Although based on a single UK programme, many constraints, such as compressed timelines and fixed scopes, are relevant to rapid evaluations internationally.

### 3.7 Implications for Future Rapid Evaluations

Our experience highlights the importance of trust‐building, responsive infrastructure, and team‐wide commitment to PPIE in rapid evaluations. Future evaluations may benefit from a whole‐programme approach, where lessons from individual projects feed into a more strategic and coherent approach that strengthen involvement over time.

Rather than applying a one‐size‐fits all model, PPIE should be tailored to the research topic, timeline, and context. Multi‐tiered structures combining standing panels, project‐specific involvement, and advisory input provide the resilience and flexibility needed for meaningful involvement in time‐sensitive environments. Future research could explore how PPIE adapts over time, including how team negotiate trade‐offs in practice, and how contributors flexibly engage at different levels depending on project needs and personal capacity.

RSET's experience offers adaptable strategies for other programmes facing similar challenges. Leveraging digital and hybrid engagement models, such as online panels and digital co‐production tools, can further enhance accessibility, efficiency, and inclusivity, making participation more feasible for diverse communities and supporting rapid integration of public insight into decision‐making.

For PPIE to be sustainable, it must be integrated into institutional policies, governance processes, and funding frameworks. This ensures that involvement is not treated as optional, but a fundamental component of high‐quality and responsive research. Strong leadership commitment and dedicated resources are also essential to underpin this work.

By reflecting on how these strategies balance what is possible, fair, and influential, this paper offers practical lessons that can be adapted to other contexts and guide thinking about feasibility and meaningfulness. Table 2 summarises eight key lessons.

**Table 2:** Lessons learned: key takeaways for PPIE in rapid research.

**Lived experience adds depth**: Include contributors from the earliest stages. **Design for inclusion**: Create flexible structures across programme and project levels. **Start early**: Plan for PPIE alongside research design. **Foster trust**: Use feedback loops and invest in relationships. **Be transparent about limits**: Acknowledge trade‐offs and co‐decide priorities. **Prioritise relationships**: Engagement is a relational, not transactional process. **Ensure leadership support**: Senior buy‐in and resourcing are essential. **Plan for continuity**: Celebrate contributions and maintain connections post‐project.

## 4 Conclusion

RSET's reflections highlight that meaningful and adaptive PPIE is achievable in rapid evaluations despite time constraints and shifting priorities. Early involvement, relational continuity, and context‐sensitive approaches required negotiating trade‐offs between feasibility, influence, and equity. These adaptations illustrate the inherent tensions in rapid evaluation, where methodological rigour, public input, and inclusivity must be balanced under time and resource constraints. A key contribution of this work is that transparent negotiation of trade‐offs is itself a marker of meaningful PPIE in rapid evaluation. Rather than viewing departures from idealised models of involvement as failures, rapid evaluations may benefit from explicitly transparent and negotiated approaches that maximise meaningful influence within practical constraints.

Trade‐offs are unavoidable, but when managed strategically and ethically, they support research that is relevant, inclusive, and impactful. Strong leadership support, adequate resources, and transparent governance were crucial in sustaining these efforts. RSET's approach offers a transferable and adaptable model for integrating meaningful PPIE in fast‐moving applied health research, ultimately contributing to work that is methodologically robust, ethically grounded, and practically actionable.

## Author Contributions

**Pei Li Ng:** conceptualisation, methodology, writing – original draft, writing – review and editing. **Raj Mehta:** conceptualisation, methodology, writing – review and editing. **Angus I. G. Ramsay:** conceptualisation, methodology, writing – review and editing, supervision. **Naomi J. Fulop:** conceptualisation, methodology, writing – review and editing, supervision. **Jenny Shand:** conceptualisation, methodology, writing – review and editing, supervision.

## Conflicts of Interest

Raj Mehta is Trustee of the Middlesex Association for the Blind (April 2015–present; Chair since December 2020); Trustee of the Research Institute for Disabled Consumers (October 2018–present; Vice‐Chair since August 2020); Non‐Executive Director of Evenbreak (February 2016–present); Trustee of the Thomas Pocklington Trust (November 2019–present). Dr Angus IG Ramsay is a Trustee of the Health Services Research UK and is supported by the NIHR Central London Patient Safety Research Collaboration. He was an associate member of the National Institute for Health and Care Research (NIHR) Health and Social Care Delivery Research (HSDR) Commissioned Board (2014–15) and associate member of the NIHR HSDR Board (2015–18). Professor Naomi J Fulop is currently a Non‐Executive Director at COVID‐19 Bereaved Families for Justice UK (from August 2022 to present), a Member of National Emergencies Trust Advisory Board (September 2025‐present), an NIHR Senior Investigator and is supported by NIHR Central London Patient Safety Research Collaboration. Professor Jenny Shand is Non‐Executive Director at Care City (June 2019–present), Advisor to the Harley Street Health District (September 2024–present), Senior Associate at ZPB Associates (February 2024–present), and an RSA fellow (December 2019–present).

## Data Availability

Data sharing not applicable to this article as no datasets were generated or analysed during the current study.
